# Barriers and facilitators of conducting research with team science approach: a systematic review

**DOI:** 10.1186/s12909-023-04619-0

**Published:** 2023-09-05

**Authors:** Arezoo Ghamgosar, Leila Nemati-Anaraki, Sirous Panahi

**Affiliations:** 1 School of Health Management and Medical Information Science, Iran University of Medical Sciences, Tehran, Iran; 2 Medical Biotechnology Research Centre, School of Paramedicine, Guilan University of Medical Sciences, Rasht, Iran; 3 Department of Medical library and Information Science,School of Health Management and Information Sciences, Iran University of Medical Sciences, Tehran, Iran

**Keywords:** Medical education, Team science, Barriers, Facilitators, Systematic review

## Abstract

**Background:**

The present review aimed to systematically identify and classify barriers and facilitators of conducting research with a team science approach.

**Methods:**

PubMed, EMBASE, PsycINFO, Scopus, Web of Science, Emerald, and ProQuest databases were searched for primary research studies conducted using quantitative, qualitative, or mixed methods. Studies examining barriers and facilitators of research with a team science approach were included in search. Two independent reviewers screened the texts, extracted and coded the data. Quality assessment was performed for all 35 included articles. The identified barriers and facilitators were categorized within Human, Organization, and Technology model.

**Results:**

A total of 35 studies from 9,381 articles met the inclusion criteria, from which 42 barriers and 148 facilitators were identified. Human barriers were characteristics of the researchers, teaming skills, and time. We consider Human facilitators across nine sub-themes as follows: characteristics of the researchers, roles, goals, communication, trust, conflict, disciplinary distances, academic rank, and collaboration experience. The barriers related to organization were institutional policies, team science integration, and funding. Organizational facilitators were as follows: team science skills training, institutional policies, and evaluation. Facilitators in the field of technology included virtual readiness and data management, and the technology barriers were complexity of techniques and privacy issues.

**Conclusions:**

We identified major barriers and facilitators for conducting research with team science approach. The findings have important connotations for ongoing and future implementation of this intervention strategy in research. The analysis of this review provides evidence to inform policy-makers, funding providers, researchers, and students on the existing barriers and facilitators of team science research.

**Trial registration:**

This review was prospectively registered on PROSPERO database (PROSPERO 2021 CRD42021278704).

**Supplementary Information:**

The online version contains supplementary material available at 10.1186/s12909-023-04619-0.

## Background

Team science is of interest in universities, organizations, funding agencies, and researchers. Scientific and societal challenges are becoming more complex, and efforts to address them require research collaborations across disciplinary, organizational, and geographic boundaries [1]. Overcoming these challenges demands a team science approach in research [2]. It represents the ability of teams to effectively collaborate as well as the capacity to integrate knowledge from diverse perspectives [3], and to answer solution-oriented research questions [4]. Team science in research is power. It has strongly facilitated the complexities of today’s research [5], and solved complex problems that researchers could not handle individually or with knowledge of a particular discipline [6, 7]. In fact, as a catalyst, team science has promoted the results of complex, new and transformed structures that are a reflection of the positive performance of multidisciplinary organizations [8].

The statistics indicate a change in the approach of researchers at the global level from individual research to team science research in all branches of science to acquire the necessary skills for their successful development and deployment [9]. The background of team science has emerged from chemistry literatures [10, 11], and was later considered by scientists studying cancer as well as nursing scientists [12–14]. Since 2004, the National Institutes of Health (NIH) established several interdisciplinary research centers and programs to promote team science [15]. In 2010, the World Health Organization (WHO) published guidelines for interprofessional collaboration, in which the implementation of clinical and research activities with team science approach was emphasized [16]. Due to its numerous advantages, team science is rapidly becoming a major practical approach among researchers as well as in biomedical research [17].

Despite increasing focus on team science approach, little is known about effective ways of collaborating through team science and across scientists, organizations, and geographic boundaries [18, 19]. Previous reviews have described a number of factors associated with effective team science and offered insight into some barriers and/or facilitators. Recently, a review found five key themes for effective implementation of team science [1]. A typology has been proposed as a basis for deriving practical guidelines for designing, managing, and evaluating successful team science initiatives [20]. A comprehensive review and practical guide to team development interventions for science teams discussed barriers to science team effectiveness, and demonstrated the deficient status of current interventions for improving science teams by applying best practices from the literature concerning teams and groups across the four phases of transdisciplinary research [21]. Implementing team science can be challenging [22]. The ways to avoid or overcome challenges have been rarely presented in the literature [20]. Therefore, to implement successful scientific collaborations with team science approach, as well as to facilitate its process, the relevant barriers and facilitators should be considered [23, 24]. Designing evidence-based approaches such as team science along with activities meant to better recognize barriers and facilitators is crucial for enhancing research outcomes.

To the best of our knowledge, no study has explored barriers and facilitators to evidence-based success and basic elements for effective teamwork in conducting research with team science and applied contexts. This paper systematically reviews the contemporary literature on a wide range of team science and employs a modified framework for the synthesis of data and the structure of barriers and facilitators. Therefore, this review aimed to synthesize evidence on the barriers and facilitators to the implementation of team science interventions in research.

## Methods

The protocol of this systematic review has been registered on PROSPERO website (www.crd.york.ac.uk/PROSPERO) (PROSPERO registration number = CRD42021278704). This systematic review protocol has been developed based on guidelines of Preferred Reporting Items for Systematic Reviews and Meta-Analyses (PRISMA) [25].

### Search strategy

The following electronic databases were searched for studies: PubMed/MEDLINE; EMBASE; PsycINFO; Scopus; Web of Science; Emerald; ProQuest. The reference lists of all the included studies were checked and scanned to identify any relevant investigations. We reviewed literature beginning from 2005/01/01 until 2023/01/01 and did not restrict the search by language. The year 2005 was chosen as the starting date because it was one year before an international conference held at National Institutes of Health. In 2006, an international conference on science of team science (SciTS) brought together experts in this nascent field to present an overview of the current state of research on team-based research and to identify priority areas and future directions for team science field [5]. An example of the full electronic search strategy for Web of Science database was as follows: (TS=(“team science”) OR TS=(“science team*”) OR TS=(“research team*”) OR TS=(“team research*”) OR TS=(interdisciplinary) OR TS=(inter-disciplinary) OR TS=(multidisciplinary) OR TS=(“multi-disciplinary”) OR TS=( crossdisciplinary) OR TS= (cross-disciplinary) OR TS=(transdisciplinary) OR TS=(trans-disciplinary)) AND (TS=(“research activities”) OR TS=(“research activity”) OR TS=( “research process”) OR TS=(“scientific process” ) OR TS=(“scholarly publish*”) OR TS=(“scientific publication*”) OR TS= (“research practice*”) OR TS=(“research study”) OR TS=(“research studies”)) AND PY=(2005–2022). The search strategies were adapted for each database to retrieve the literature related to team science approach. Duplicated citations were removed using EndNote X8 software, and a manual revision was done for verification.

### Inclusion and exclusion criteria

This systematic review included all primary research studies conducted using quantitative, qualitative, or mixed methods, which were relevant to the purpose of this study and investigated barriers and facilitators of conducting research with a team science approach. We focused on team science studies suggested by NIH Field Guide Definition. We restricted our attention to team research involving contributions and continuing collaboration by scientists who represent at least two separate disciplines as they address together a research question [26]. Studies examining barriers and facilitators of conducting research with a team science approach were included. Those focusing on healthcare, hospitals, clinical teams, care settings, sports teams in non-science settings, and so forth were excluded. Resources of other types such as reviews, systematic reviews, editorials, letters to editors, reports, notes, short communications, and patents were also excluded.

### Study selection

Two reviewers (AGh, SP) independently screened titles and abstracts of articles. They then independently selected the full text of primary studies from the first screening phase and reported the reasons for the exclusion of articles. Discrepancies between authors at any stage were resolved via consensus between the two reviewers, and when this was not sufficient, they discussed the matter with a third reviewer (L N-A) whose decision was finalized.

### Data extraction

Data were identified and extracted from each primary study independently by two reviewers (AGh, SP) using a data extraction form. We restricted our attention in this paper to team research involving contributions and continuing collaboration by scientists who represent at least two separate disciplines as they address together a research question. The information we extracted involved the first author, title, study setting and design, year of publication, country, sample size, participants, and findings (outcomes). The barriers and/or facilitators in the way of team science approach, as well as the interpretation of results were extracted. If an item in the text of the study was considered a barrier, it was classified in the section of barriers, and if an item was a facilitator, it was grouped in the section of facilitators. Inconsistencies were settled through consensus between the two reviewers, and in case it did not work, a third reviewer (L N-A) was consulted whose judgment was finalized.

### Quality assessment of the studies

Using the Mixed-Methods Appraisal Tool (MMAT), the quality of the included articles was independently evaluated by two authors (AGh and SP). MMAT has been developed to enable quality assessment of different study designs using a single tool involving various criteria for articles reporting quantitative, qualitative, and mixed-method studies. This tool includes two screening questions, as well as five questions per study design, in which response options are ‘yes’, ‘no’, and ‘can’t tell’. The ‘can’t tell’ response category indicates that the article does not report appropriate information to answer ‘yes’ or ‘no’ or that it reports unclear information related to the criterion [27]. For our review, questions concerned with descriptive, quantitative, qualitative and mixed-method studies were considered. Disagreements were decided through compromise between the two reviewers, and in case this was not useful, we referred to a third reviewer (L N-A) to finalize the decision. All articles were deemed to be of sufficiently high quality and were included in this research.

### Data synthesis

Although several conceptual model frameworks are available for conducting and implementing science [28], none of them covers all aspects of scientific collaborations with a team science approach. Team science initiatives normally require human, organizational, and technology resources. Thus, one of these frameworks, namely the Human, Organization, and Technology-fit (HOT-fit) model [29], was chosen and modified to classify barriers and facilitators into themes. We used the HOT-Fit framework as the main structure and theme of our study; we then placed the sub-themes resulting from the data synthesis of the selected articles in the relevant section of synthesizing evidence about the barriers and facilitators of the use of team science in research. This assessment model defines the components of an information system as the main components to be evaluated, namely the human, organizational, and technological components as well as the relationship between these components [29]. Data analysis and grouping was done independently using inductive method by (AGh, SP). The codes were generated based on statements, words, descriptions and concepts expressed in the text. Afterward, semantic units with similar content received the same code. In the following, these codes were grouped into sub-subthemes based on their differences or similarities. Sub-subthemes with similar meaning and concept were regrouped into subthemes. Eventually, by comparing the subthemes, the main theme was obtained. At this stage, any disagreements were resolved through discussion and exchange of opinions between authors (AGh, SP). Also, an external subject-expert (L N-A) who had a history of conducting qualitative research reviewed and approved the coding process and categories. Excel software was used to analyze and interpret the data. We synthesized the results by content analyses because heterogeneity between studies impeded the pooling of data in a meta-analysis.

## Results

**Fig. 1:**
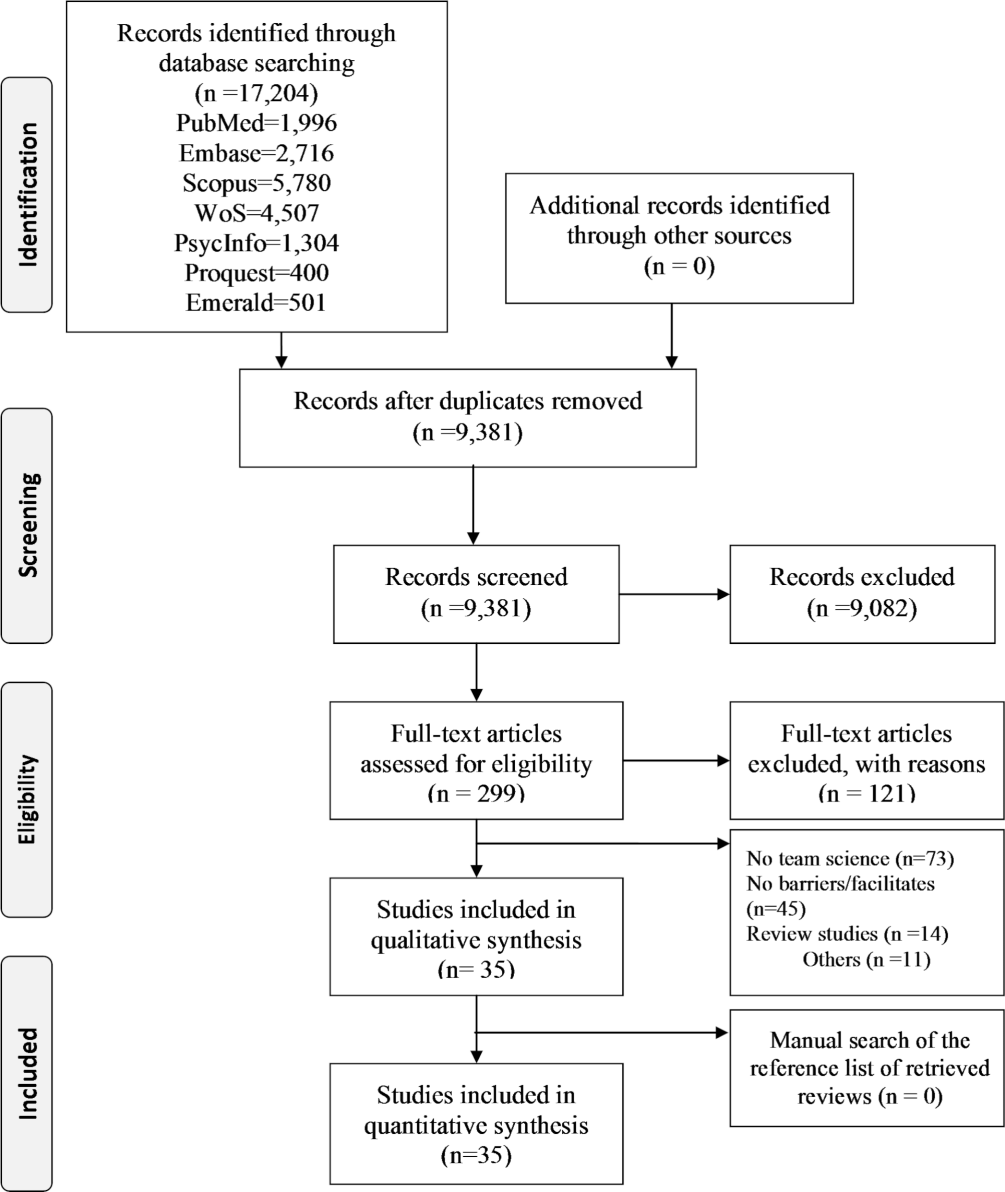
PRISMA flow diagram of study identification

Figure 1 shows the flowchart of selecting the studies. A total of 17,204 records were identified through searching the seven databases. After eliminating the duplicates, 9,381 unique records were screened for title and abstract in terms of eligibility, of which 299 original articles were included in the full-text screening. Finally, 35 articles were identified for inclusion in this review.

### Trends and characteristics of articles

**Fig. 2:**
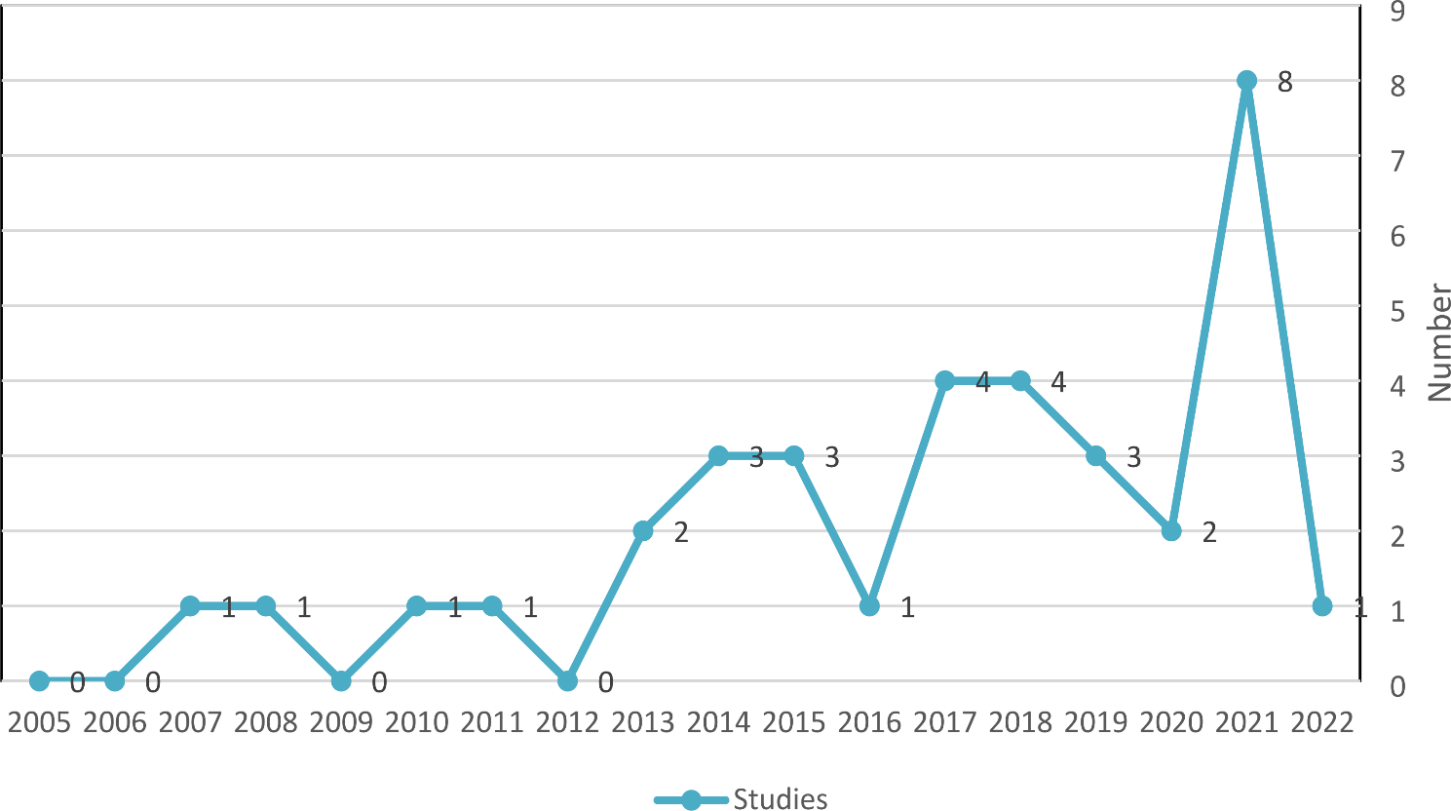
The published studies by year

The included articles were published between 2007 and 2022, with a majority of them (n = 8) published in 2021 (see Fig. 2).

The universities (n = 33) and scientific meetings (n = 2) were the most frequent setting of studies. Most studies included in the review were published in high-income countries, with the majority conducted in USA (n = 27), followed by Canada (n = 2), Sweden (n = 2), Australia (n = 1), UK (n = 1), Italy (n = 1) and China (n = 1). Only one study from China (as a developing country) investigated the barriers and facilitators in team science, and the rest of studies originated from developed countries. Fourteen articles employed qualitative designs, twelve articles used quantitative designs, and nine articles took advantage of mixed-method designs. Participants were faculty members, fellows, stakeholders, researchers, education leaders, students, directors, nurses, investigators, and trainees. Six articles described both barriers and facilitators to conducting research with team science approach [30–35], while twenty-four articles described only facilitators [36–60] and four articles defined only barriers [61–64]. Table 1 shows a description of the included studies examining the most frequent barriers and facilitators of conducting research with team science approach.

**Table 1:** Characteristics of the included studies

Author, Year, Country, (Reference)	Title	Context, Setting	Study Design or Focus/tools	Participants/sample size	Barrier or facilitator
Aarons GA, 2020, USA, [36]	Identifying strategies to promote team science in dissemination and implementation research	University	Qualitative study utilizing semi-structured interviews	27 participants – Faculty and fellows	Facilitator
Allen ML, 2010, USA, [37]	Facilitating research faculty participation in CBPR: Development of a model based on key informant interviews	University	Qualitative study utilizing semi-structured interviews	13 participants-Faculty	Facilitator
Arevian AC, 2018, USA, [61]	Participatory methods to support team science development for predictive analytics in health	A scientific symposium	Quantitative study utilizing a survey.	85 participants - stakeholders	Barrier
Ayre M, 2015, Australia, [38]	Doing integration in catchment management research: Insights into a dynamic learning process	University	Qualitative study utilizing interview.	50 participants -researchers	Facilitator
Begg MD, 2014, USA, [39]	Approaches to preparing young scholars for careers in interdisciplinary team science	University	Quantitative study utilizing a survey	60 participants –Education leaders	Facilitator
Behar-Horenstein LS, 2017, USA, [40]	Exploring mentoring in the context of team science	University	Qualitative study utilizing semi-structured interviews.	10 participants-faculty mentors and postdoctoralmentees	Facilitator
Blakeney EAR, 2021, USA, [41]	Implementation and evaluation of team science training for interdisciplinary teams in an engineering design program	University	Mixed methods study combining qualitative methods utilizing semi-structured interviews and a quantitative tool	100 participants- students	Facilitator
Bridle H, 2013, Italy, [42]	Preparing for an interdisciplinary future: A perspective from early-career researchers	A scientific meeting	Quantitative study utilizing a survey	34 participants-early-career researchers	Facilitator
Brower HH, 2021, USA, [43]	Creating effective academic research teams: Two tools borrowed from business practice	University	Mixed methods study combining qualitative methods utilizing semi-structured interviews and a quantitative tool	50 participants-research scientists	Facilitator
Christensen J, 2021, Sweden, [62]	The beautiful risk of collaborative and interdisciplinary research. A challenging collaborative and critical approach toward sustainable learning processes in academic profession	University	Mixed methods study combining qualitative methods utilizing interviews and a quantitative tool	85 participants-faculties	Barrier
Cosley BJ, 2014, USA, [34]	Collaborative Voice: Examining the Role of Voice in Interdisciplinary Collaboration	University	Quantitative study utilizing a survey	27 participants-faculties	Barrier and facilitator
DeHart D., 2017, USA, [30]	Team science: A qualitative study of benefits, challenges, and lessons learned	University	Qualitative study utilizing semi-structured interviews.	9 participants-research scientists	Barrier and facilitator
Gavens L., 2018, UK, [31]	Interdisciplinary working in public health research: a proposed good practice checklist	University	Qualitative study utilizing semi-structured interviews	20 participants-senior professors, researchers	Barrier and facilitator
Guise JM, 2017, USA, [44]	Team Mentoring for Interdisciplinary Team Science: Lessons From K12 Scholars and Directors	University	Quantitative study utilizing a survey	78 participants-directors and active and former scholars	Facilitator
Guise JM, 2017, USA, [45]	Organizational and training factors that promote team science: A qualitative analysis and application of theory to the National Institutes of Health’s BIRCWH career development program	University	Qualitative study utilizing interviews	NA- participants-Research Careers	Facilitator
Hebert-Beirne J, 2021, USA, [46]	Novel (Multilevel) Focus Group Training for a Transdisciplinary Research Consortium	University	Mixed methods study combining qualitative methods utilizing interviews and a quantitative tool	15 participants-Researcher	Facilitator
Hellström T, 2018, Sweden, [47]	Governing interdisciplinary cooperation in Centers of Excellence	University	Qualitative study utilizing semi-structured interviews	40 participants-directors	Facilitator
Love HB, 2021, USA, [48]	Interpersonal relationships drive successful team science: an exemplary case-based study	University	Mixed methods study combining qualitative methods utilizing interviews, social network surveys, participant observation, focus groups, and a quantitative tool	Participants (25 teams)- Principle Investigators, postdoctoral researchers, students, and active collaborators	Facilitator
Luo J, 2022, USA, [49]	Relationships between changing communication networks and changing perceptions of psychological safety in a team science setting: Analysis with actor-oriented social network models	University	Quantitative study utilizing a survey	64 scholars	Facilitator
Mayowski CA, 2019, USA, [50]	Developing a team science workshop for early-career investigators	University	Mixed methods study combining qualitative methods utilizing interviews and a quantitative tool	30 participants- Early career investigators	Facilitator
McCormack WT, 2021, USA, [51]	CTS teams: a new model for translational team training and team science intervention	University	Quantitative study utilizing a survey	58 participants- pre-doctoral students and co-mentors	Facilitator
Milman A, 2015, USA, [63]	Scholarly motivations to conduct interdisciplinary climate change research	University	Quantitative study utilizing a survey	526 participants- Ph.D.	Barrier
Morse WC 2007, USA, [52]	Bridges and barriers to developing and conducting interdisciplinary graduate-student team research	University	Qualitative study utilizing semi-structured interviews	18participants- faculty and students	Facilitator
Nair KM, 2008, Canada, [32]	It’s all about relationships: a qualitative study of health researchers’ perspectives of conducting interdisciplinary health research	University	Qualitative study utilizing semi-structured interviews	19 participants- health researchers	Barrier and facilitator
Norman MK, 2018, USA, [53]	The teams of early-career investigators: a qualitative pilot study	University	Qualitative study utilizing semi-structured interviews	22 participants- leaders and junior faculty	Facilitator
Puga F, 2013, USA, [54]	The teams of early-career investigators: a qualitative pilot study	University	Mixed methods study combining qualitative methods utilizing interviews and a quantitative tool	200 participants-Nurses, coordinator, Physician, Educator, Faculty	Facilitator
Read EK, 2016, USA, [55]	Building the team for team science	University	Quantitative study utilizing a survey	44 participants-early career scientists	Facilitator
Roelofs S, 2019, Canada, [35]	Formative, embedded evaluation to strengthen interdisciplinary team science: Results of a 4-year, mixed methods, multi-country case stud	University	Mixed methods study combining qualitative methods utilizing interviews and a quantitative tool	100 participants-project coordinator, secretary, and all research team members	Barrier and facilitator
Salazar M, 2011, USA, [56]	To join or not to join: an investigation of individual facilitators and inhibitors of medical faculty participation in interdisciplinary research teams	University	Quantitative study utilizing a survey	828 participants- faculty	Facilitator
Tkachenko O, 2020, USA, [59]	Critical factors impacting interdisciplinary university research teams of small size: A multiple-case study	University	Qualitative study utilizing semi-structured interviews	12 participants- faculty and student researchers	Facilitator
Turner VK, 2015, USA, [64]	Essential tensions in interdisciplinary scholarship: navigating challenges in affect, epistemologies, and structure in environment–society research centers	University	Qualitative study utilizing semi-structured interviews	18 participants- faculty	Barrier
Vaughan R, 2021, USA, [57]	The Rockefeller Team Science Leadership training program: Curriculum, standardized assessment of competencies, and impact of returning assessments	University	Mixed methods study combining qualitative methods utilizing interviews and a quantitative tool	15 participants- the Scholar’s primary mentor and senior staff	Facilitator
Vogel AL, 2014, USA, [33]	Pioneering the Transdisciplinary Team Science Approach: Lessons Learned from National Cancer Institute Grantees	University	Qualitative methods utilizing interviews and a quantitative tool	31 participants- investigators and trainees	Barrier and facilitator
Wallen KE, 2019, USA, [60]	Integrating team science into interdisciplinary graduate education: an exploration of the SESYNC Graduate Pursuit	University	Quantitative study utilizing a survey	39 participants- doctoral student or candidate	Facilitator
Zhang X, 2021, China, [58]	Team learning in interdisciplinary research teams: antecedents and consequences	University	Quantitative study utilizing a survey	304 participants- Researcher	Facilitator

Quality ratings have been reported in an additional file 1. All studies had a clear statement of research questions and designs. Ten out of fourteen articles reporting qualitative findings rated ‘yes’ for all seven related items [30, 32, 33, 36, 37, 40, 47, 52, 53, 59]. Comparatively, eight out of twelve articles reporting quantitative descriptive findings received a ‘yes’ grade for all the relevant seven items [39, 45, 49, 51, 56, 58, 60, 63]. From nine articles that reported mixed-method findings, no article rated ‘yes’ for any of the 17 related items. (see Additional file 1).

### Barriers and facilitators

In 35 included studies, 42 barriers as well as 148 facilitators were recognized. To better understand the factors affecting research with team science approach, HOT-fit framework involving Human, Organization, and Technology themes was used and modified. HOT-fit framework is divided to domains related to Human, Organization, and Technology, each with several subthemes to highlight various levels of the factors (barriers and facilitators). A list of identified barriers and facilitators is shown in Table 2. Comprehensive list of identified barriers and facilitators related conducting research with team science approach based on the HOT-fit framework is shown in additional file 2. (see Additional file 2). A conceptual model was plotted based on themes and sub-themes that show the how the barriers/facilitators are interrelated. (see Additional file 3). Several barriers and facilitators were unique and unexplored in the literature (e.g., faulty assumptions regarding team members’ skills and compliance with good clinical practice), whereas others were replications of each other (e.g., motivation and communication). The evaluation was a factor that previously received only limited attention; however, our study showed that it substantially influenced research teams. What follows is a review of the key factors and their characteristics.

### Human

Barriers and facilitators were identified from the Human component of HOT-fit framework. Human barriers across the three sub-themes were characteristics of the researchers (n = 6), teaming skills (n = 11), and time (n = 8). We consider Human facilitators across nine sub-themes as follows: characteristics of the researchers (n = 9), roles (n = 24), goals (n = 15), communication (n = 25), trust (n = 6), conflict (n = 9), disciplinary distances (n = 8), academic rank (n = 3), and collaboration experience (n = 3). The most frequently identified human barriers were considerations related to teaming skills, and the most commonly identified human facilitator was communication.

### Organization

Barriers and facilitators were identified from Organization component of HOT-fit framework. The barriers related to Organization category were divided into factors such as institutional policies (n = 7), team science integration (n = 5), and funding (n = 8). We consider organization facilitators across three sub-themes as follows: team science skills training (n = 21), institutional policies (n = 11), and evaluation (n = 7). Team science skills training was identified as the most pervasive barriers in this domain.

### Technology

The recognized facilitators in the technology theme included virtual readiness (n = 1), and data management (n = 1), and the identified technology related barriers were complexity of techniques (n = 1), and privacy issues (n = 2).

**Table 2:** A list of identified barriers and facilitators related to conducting research with team science approach based on HOT-fit framework

Barriers: Themes and subthemes (References)	Facilitators: Themes and subthemes (References)
**Themes 1: Human** **Sub-theme1**: Characteristics of researchers [33, 42, 56, 60, 62] **Sub-theme 2**: Teaming skills [34, 42, 51, 56, 59, 60, 62] **Sub-theme 3**: Time [31, 34, 37, 42, 47, 56, 62, 63]	**Themes 1: Human** **Sub-theme 1**: Characteristics of researchers [33, 36, 48, 49, 56, 57, 60] **Sub-theme 2**: Roles [32, 36, 41, 43, 44, 47, 48, 52, 56, 57, 60, 62–64] **Sub-theme 3**: Goals [31, 36, 38, 43, 44, 47, 48, 52, 53, 56, 57, 63, 64] **Sub-theme 4**: Communication [32, 33, 36, 38, 41, 43, 44, 46, 47, 52–54, 56, 60, 62–64] **Sub-theme 5**: Trust [32, 38, 41, 48, 52, 64] **Sub-theme 6**: Conflict [33–35, 38, 41, 48, 52, 63, 64] **Sub-theme7**: Disciplinary distances [31, 38, 41, 44, 55, 56, 64] **Sub-theme 8**: Academic Rank [55, 56] **Sub-theme 9**: Collaboration Experience [52, 55, 56]
**Themes 2: Organization** **Sub-theme1**: Institutional policies [37, 42, 51, 57, 59] **Sub-theme2**: Team Science Integration [37, 57] **Sub-theme3**: Funding [31, 37, 51, 56, 62, 64]	**Themes 2: Organization** **Sub-theme1**: Institutional policies [31, 35, 36, 41, 44, 47, 56, 62] **Sub-theme2**: Team Science skills training [30, 31, 34, 36, 38, 39, 41, 43–46, 48, 50, 53, 54, 61, 63, 64] **Sub-theme3**: Evaluation [32, 44, 48, 60, 63, 64]
**Themes 3: Technology** **Sub-theme1**: Complexity of techniques [37] **Sub-theme2**: Privacy issues [37]	**Themes 3: Technology** **Sub-theme1**: Virtual readiness [53] **Sub-theme2**: Data management [64]

## Discussion

This systematic review aimed to identify barriers and facilitators to the implementation of team science interventions in research. We identified three barriers and nine facilitators into Human theme, three barriers and three facilitators into Organization theme, and two barriers and two facilitators into Technology theme. The findings of this review were consistent and closeness with a study that examined the complement of intrapersonal, organizational, institutional, physical, environmental, and technological, as well as political and societal factors influencing the efficiency of transdisciplinary collaboration in research teams [20]. In another study, the empirical evidence and research gaps of collaboration in science were grouped into the following five themes: the value of team science, team composition and its influence on team science performance, formation of science teams, team processes central to effective team functioning, and institutional influences on team science [1]. However, in our investigation, a number of (sub-) themes have been defined, which provide more details on barriers and facilitators by comparing with the two previous studies. Unlike the two mentioned studies, our research was a systematic review that examined a wide range of scientific literatures, and focused on team science studies. The details of the findings of this study are discussed below.

### Human

Factors in this domain were highly cited across all papers. The analysis of extracted factors showed that most barriers and facilitators should be tackled by human. As a result, in the human theme, the facilitators related to “communication” and “roles” sub-themes were the most frequent. **Barriers.** “Different philosophies and styles” in research [30, 31], “traditional views on research” [59] and “lack of correct understanding of team science research” [59] will lead to challenges in research with team science approach. It is suggested to check these characteristics by interviewing potential participants when forming a team. The most common obstacle identified in the human theme was considerations related to team forming skills. Some researchers may be “less engaged in research” [59], and others may have “wrong assumptions and ideas about their skills” [59] and not be able to perform the required tasks, which in both cases will delay research. These challenges can be resolved to some extent by “defining each person’s role” and “claiming responsibility” [58] through “strong leadership” [32]. One risk identified as a barrier was that some specialties were represented by only one expert and researcher in the group, meaning that if that expert left the group, the research process would be hampered. “Recruiting senior and junior researchers” [31] together in each discipline will help maintain interdisciplinary interaction and increase staff flexibility. It is recommended to focus on the challenges related to team building skills to set sustainable strategies during team formation.

Team science is a time-consuming task. “Diversity in the background, research culture, and discipline” [42, 47, 52] may require longer coordination time among group members. Adequate time must be allocated to overcome the challenges of “working with multiple researchers” [30]. Therefore, it is of high importance to consider a practical scope and time frame to overcome the challenges of team science work. In the early stages of team formation, it is necessary to create a timeline as well as a research framework that specifies the responsibilities of each team member. It is suggested that a long period of time be considered according to the need for conducting this type of research, as well as allocating more time and effort to coordinate and carry out the assigned responsibilities at due time.

**Facilitators.** Researcher’ personality traits such as “motivation”, “mental health” [49, 59] and “interpersonal skills” [59, 60] influence team science research teams. People having “positive attitudes and beliefs” [33, 37] toward team science should be invited to the team, those who are “willing and eager to cooperate” [37], and observe “mutual respect” [50] must be invited to the team. Individuals who can share a “common mental model” with group members may improve team performance [48, 58, 59]. It is better to explain the research objectives to all researchers after selecting the team members and determining the arrangement of the team.

The leader and each member of the group should be selected with “thinking and strategy” [50, 52, 57] through “interview” [31, 50, 57, 59]. To integrate disciplines and bring them closer together, a “common mental model” should be presented in research with a team science approach [31]. Experienced researchers invite “young researchers” to join their projects [59]. “The roles and responsibilities of each member of the group” must be defined for all stakeholders and organizations [32, 33, 35, 36, 41]. Assigning roles is a factor often mentioned in team science texts that indicates its importance.

Conducting research with a team science approach is a complex process. Clear “goal setting” is a key strategy in doing research [43, 53]. Also, “the definition of the objectives” of the study and research questions should be clearly expressed [42, 43, 52].

The results indicated that the main theme of “communication” was the most important theme related to the human. Scientific progress in research with team science approach has significant advantages when there is communication between scientists of different disciplines. Communication facilitates researchers’ interest in contributing to the group [34, 48]. The link between researchers plays an essential role in the development and implementation of their research [55, 59]. The presence of effective communication helps clearly define roles, establish “trust” and “efficient working relationships” among team members [58, 60]. “Face-to-face communication”, “communication platforms” and “online tools” were proposed in order to establish communication in this regard [43, 54]. None of the selected papers mentioned communication through conventional platforms, while today these tools and programs play a major role in communication.

Conflict was identified as the main theme affecting research with team science approach. Organizations need practical skills training in areas such as conflict management [50, 57, 60]. When conflicts are anticipated and subsequently managed, team science research results are expected to yield tangible outcomes. In team science texts, conflict management is mentioned, but no specific plan and strategy is provided for it. It seems that further research is necessary on conflict management in team science.

Successful implementation of modern medical research on complex phenomena requires different expertise. The nature of team science is currently a special approach with the cooperation of different specialties. Solutions are provided in the texts to approximate different fields. The “semi-formal organizational structure” of team science can address the challenges that researchers and institutions face while conducting and supporting team science [45, 47]. These semi-formal structures can act as a bridge enabling communication between different organizations, universities and researchers [45].

When conducting research with a team science approach, several other strategies regarding member selection, team composition and team building should be considered, including the “academic degree” criterion. Some medical professionals are more inclined to participate in research than others. Specifically, basic science researchers, associate professors, and faculty members with distinct subject matter expertise and “prior collaboration experience” are more likely to participate in research relative to their peers [53, 56, 58, 59]. Factors like “previous work-related experience” influence the choice of participation in a science team. Studies have shown that researchers with prior collaboration experience are more disposed to join research with team science approach [56]. One of the reasons for this may be the researchers’ lived experience of joining this type of research. “Previous scientific output” of researchers should be considered when forming teams [59]. If possible, those who have prior scientific cooperation experience should be invited to the team because experienced people contribute to team dynamics.

### Organization

As a result, in the organization’s theme, the facilitator related to “team science skills training” sub-theme was the most frequent. **Barriers.** Organizational policies play an important role for researchers in choosing team science research in the future, and “lack of organizational support” was mentioned as an obstacle [63]. Some organizations with “traditional views” do not recognize or reward team science [33]. It seems that the adoption of supportive policies by organizations to uphold team science research is a facilitating factor.

Before researchers begin research with team science approach, they need to fully understand “discipline-based differences” through education. Including the training of team science approaches in educational planning and activities may be an effective model for involving scientific fields and stakeholders in research [61]. “Differences in disciplines, terms, methods and working styles” can lead to misunderstandings or conflicts [33]. To create a bridge between researchers, we suggest that organizations consider methods such as holding workshops to promote collaboration and strengthen team science.

The shortage of funds for scientific cooperation is among the organizational obstacles [61, 63]. Providing financial resources facilitates and supports the implementation of team science research [33, 47, 57, 59]. Organizations providing financial resources can help improve team science through providing and allocating financial opportunities [33] because the availability of potential financial resources affects the choice of projects. Considering the necessity of conducting research with team science approach, the allocation of more stable resources will help introduce and promote research as well as encourage researchers.

### Facilitators

Lack of attention to any of the effective organizational factors can lead to the failure of research adopting a team science approach. “Organizations that have a positive attitude towards cooperation” [31, 37] develop organizational structures to support research with team science approach [47, 59]. “Supportive policies of the organization” is another organizational factor that contributes to participation in team science [31, 37, 45]. Planning by organizations to “hold educational workshops” for coordination between different disciplines and specialties and to clarify the differences in this field is of great importance [38, 41, 47]. Organizations should define common team “goals” and mental models for different disciplines [33, 36, 42, 43, 45, 50, 52–54, 57] to minimize the differences.

Teaching team science skills [39, 41, 43, 46–48, 50–52, 55, 56], hiring “team-science coaches” [40, 44, 45] and “training sessions” will improve group performance [54]. Educators should “educate the group” by holding training workshops [30, 40, 44, 57]. Most researchers are not trained in “team science” skills [43]. Therefore, developing educational programs for researchers and even involving them in curricula can prepare researchers for the production of organized science in the future.

Formal evaluation processes are a mechanism for organizations as well as a program to support team science [45]. Therefore, different organizations develop an “accountability strategy” for assessment, and a strategy that helps team members clarify the “team science timeline” as well as the requirements and responsibilities for everyone [38, 44, 48, 60]. However, it should be emphasized that the evaluation of research with team science approach is an evolving and challenging field [48], and all factors affecting team science should be considered in the evaluation processes.

### Technology

Implementing a robust technical infrastructure to support long distance collaboration [54] and considering “websites for the research team” to hold training sessions for participating in collaborative research [54] were facilitators of team science. The websites provide a variety of technical resources, including shared “web space, access to conference lines, and a centralized database” [40], that is, they provide a stable technical infrastructure to support virtual collaboration, promote readiness to collaborate, and supervise data management, “data security, and data sharing” [54, 57]. Only one study focused on barriers to technology. “Complexity of techniques, data sharing and privacy issues” were the key challenges in technologies, analytic techniques, and merging of large data sets [61]. Having a plan to address barriers to technology should be considered as one of the concerns for the organizations. Technology is an important topic that is not generally well covered in the literature.

## Conclusions

The findings of this study showed that the implementation of team science research process can be influenced by the dimensions of that exist within human relationships, organizational structures, and technological infrastructures. Considerations of how these dimensions influence each other can guide future direction and implementation of team science research efforts. Attention to these themes and sub-themes when implementing and developing team science intervention could facilitate team efficiency. Despite the potential of team science in promoting science, the influence of the technology for its successful implementation in research has not been sufficiently investigated and requires further exploration. Research results at both individual and organizational levels can support the implementation of team science research. Scientific teams face many challenges due to their multifactorial nature. Identifying the challenges will help policymakers, institutions, funders, and researchers make successful decisions. Identifying the benefits will aid them in implementing and participating in teams knowledgeably. According to the results of the present study, a conceptual framework can be designed as a guide for the successful implementation of science teams.

### Strength and limitations

The current study is the first systematic review evaluating the barriers and facilitators to team science implementation in research settings from the perspective of multiple stakeholders including faculty, fellows, stakeholders, researchers, education leaders, students, directors, nurses, investigators, and trainees. This research has some limitations that should be mentioned. The strength of our study was the large number of included studies, which provided strong evidence on the barriers and facilitators of conducting research with a team science approach. Despite searching and reviewing a large number of studies from seven databases, some studies have not been retrieved and reviewed due to human false.

### Electronic supplementary material

Below is the link to the electronic supplementary material.

Supplementary Material 1

Supplementary Material 2

Supplementary Material 3

## Data Availability

The datasets used and/or analyzed during the current study are available from the corresponding author on reasonable request.
