# Correction: Barriers and facilitators of conducting research with team science approach: a systematic review

**DOI:** 10.1186/s12909-023-04705-3

**Published:** 2023-09-29

**Authors:** Arezoo Ghamgosar, Leila Nemati-Anaraki, Sirous Panahi

**Affiliations:** 1 School of Health Management and Medical Information Science, Iran University of Medical Sciences, Tehran, Iran; 2 Medical Biotechnology Research Centre, School of Paramedicine, Guilan University of Medical Sciences, Rasht, Iran; 3 Department of Medical library and Information Science, School of Health Management and Information Sciences, Iran University of Medical Sciences, Tehran, Iran

**Correction: BMC Medical Education (2023) 23:638**

10.1186/s12909-023-04619-0

Following publication of the original article [1], the authors corrected the first affiliation.

The affiliation has been updated above and the original article has been corrected.
