# Supplementary material for: Barriers and facilitators of conducting research with team science approach: a systematic review
Source: BMC Med Educ. 2023 Sep 5;23:638. doi: 10.1186/s12909-023-04619-0 (PMC10478305; doi:10.1186/s12909-023-04619-0)
Supplement: Supplementary file 1 — Supplementary Material 1 [file 12909_2023_4619_MOESM1_ESM.docx]

**Additional file 2** A comprehensive list of identified barriers and facilitators related conducting research with team science approach based on the HOT-fit framework.

| Barriers: Themes and subthemes (References) | Facilitators: Themes and subthemes (References) |
| --- | --- |
| **Themes 1: Human**  **Sub-theme1:** Characteristics of researchers  -Divergent philosophies and styles  - Traditional perspectives about what makes a successful scientist  - Not understanding what interdisciplinary research is really about. [33, 42, 56, 60, 62]  **Sub-theme 2:** Teaming skills  - Fit researchers within academic context  -The tenure and promotion criteria for newer investigators  -Roles and responsibilities of participants  -Staffing resilience  -Marginality and power dynamics  - Faulty assumptions about team members’ skills  -The challenge of knowing the not-known  - Tensions for individual researchers  - The challenge of forming common conceptual knowledge  -Challenges in communication across disciplines  -Lack of peer support [34, 42, 51, 56, 59, 60, 62]  **Sub-theme 3:** Time  -Excessive time/effort required  -The challenge of taking time  - Busyness (researchers)  -Length of the encounter (Time)  -Timeframe  -Team science takes time  - Integrated work may take longer  [31, 34, 37, 42, 47, 56, 62, 63] | **Themes 1: Human**  **Sub-theme 1:** Characteristics of researchers  -Beneficial attitudes and beliefs about transdisciplinary research and team science  - Ready and eager to collaborate  -Maintaining mutual respect  -Motivation  -Psychological safety  -Interpersonal skill  -Active leadership by principal investigator to keep all disciplines engaged and contributing  [33, 36, 48, 49, 56, 57, 60]  **Sub-theme 2:** Roles **(**Defines the team’s roles)  - Select team members thoughtfully and strategically  -Staffing  -Group size  -Being an effective team leader  -Career stage of participants: PhD, MS, MD students  -Define team roles and responsibilities for different types of members (researchers, trainees, and project staff) and organizations  - Clarifying tasks  -Clarifying roles and expectations  -A plan for disciplinary integration  -Role clarity in mentoring  -Brokering and bridge-building activities by individuals holding particular roles in a research center  - Clearing team membership with Overlapping Groups and Lines of Affiliation Often Running Through the principal investigator (Roles)  - An explicitness about the role and contribution of each discipline  -Recognize team members' strengths  -Holding team members accountable  -Compliance with Good Clinical Practice  [32, 36, 41, 43, 44, 47, 48, 52, 56, 57, 60, 62-64]  **Sub-theme 3:** Goals  -Establishing shared goals and mission  -Defines the team’s purpose,  -Defines the team’s strategies  -Need for a theme or topical focus  -Define focal themes and research questions jointly and clearly  -Emphasize problem definition and team proposal writing  -Shared context and goals  -Developing shared goals  -Establishing explicitly stated, high-level group goals, relying on the grant for direction and focusing on immediate task goals (Goals)  -Shared mental model  -Creating and communicating a vision  - Effective team processes (goals)  -Shared interests and complementary expertise  -Shared purpose  -Shared mental model team performance [31, 36, 38, 43, 44, 47, 48, 52, 53, 56, 57, 63, 64]  **Sub-theme 4:** Communication  -Promoting and modeling effective communication  -Communication with team members outside of class  - Open communication across researchers from different fields  - Develop formal and informal communication strategies  -Regular interaction/communication by team members  - Highly valuing by responsiveness to communication and the free flow of information within teams  - Building communication competence  -Establishing lines of open communication  - Interactions: Inclusive approach to face-to-face meetings to develop good working relationships)., Friendly atmosphere, Lively discussion  -Developing good relationships between researchers from different disciplines  -Meetings to promote communication  -Use of tools for facilitating team effectiveness/organization  - A protocol implementation kit to facilitate study management  -Effectively contribute in team meetings  -Speak up in team meetings  -Determine a Location of meeting  -Demonstrations of disinterest, location of experts  - Using upcoming face-to-face annual meeting  - Importance of voice for interpersonal relationships among co-workers  - Interpersonal relationships: learning, leadership, mentoring, advice, friendship, and having fun  -Building professional relationships  - Communication and teamwork  -Coordination [32, 33, 36, 38, 41, 43, 44, 46, 47, 52-54, 56, 60, 62-64]  **Sub-theme 5:** Trust  - A high level of trust between team members  -Trusting  -Trusting with team members  -Maintaining team morale  -Building trust among team members [32, 38, 41, 48, 52, 64]  **Sub-theme 6:** Conflict  - Team members not including disagreements about science or roles in their definition of conflict  -Constructive conflict  - Address temporal and spatial scale issues  - Resource-Constraining by teams, with the potential for tension and conflict as a result  -Conflict resolution  - Providing and integrating a planned ‘device for the possibility of translation  -Resolving conflicts with peers and other project collaborators. [33-35, 38, 41, 48, 52, 63, 64]  **Sub-theme7:** Disciplinary distances  - Having collaborated to make their voice heard in team meetings  -Advocate for multiple points of view  -Closing of disciplinary distances among center participants  -Supporting an informal epistemic environment  - Semiformal meta-organizational structure  -Compliance with federal and state laws and regulations  -Distinctive expertise: medical faculty with distinctive topic area expertise were more likely to join an interdisciplinary research team  - Balance in team members’ expertise and input  - Task interdependence  [31, 38, 41, 44, 55, 56, 64]  **Sub-theme 8:** Academic Rank  - Type of work researcher: a basic researcher greater rate than their peers for a team science approach  -Academic rank: the associate professor rank may be greater rate than their peers for participating in an interdisciplinary research  - Member status [55, 56]  **Sub-theme 9:** Collaboration Experience  - Medical experts with greater experience collaborating across departmental boundaries were more likely to join an interdisciplinary research team  - Previous and intermediate outputs  - Early-Career investigators appeared uncomfortable assuming a leadership role. [52, 55, 56] |
| **Themes 2: Organization**  **Sub-theme1:** Institutional policies  -Lack of institutional support and promotion requirements  -Structural tension  - The challenge of creating a learning environment  -Traditional incentive and reward systems that do not recognize or reward transdisciplinary team science  -Distance or other physical barriers  - Limited published guidance for how to engage in transdisciplinary team science  -Longer time frame for conducting projects, and publishing [37, 42, 51, 57, 59]  **Sub-theme2:** Team Science Integration  -Discipline-based differences in values, terminology, methods, and work styles  -Conceptual and scientific challenges inherent to efforts to achieve transdisciplinary integration  -project management challenges involved in transdisciplinary team science  -Identifying individuals in other areas  -Lack of perceived need to collaborate by others [37, 57]  **Sub-theme3:** Funding  -Lack of funding for collaborations  -Allocating extra resource  -Appropriate budgeting  - supporting transdisciplinary team science by Funding initiative characteristics  -Funding [31, 37, 51, 56, 62, 64] | **Themes 2: Organization**  **Sub-theme1:** Institutional policies  -Having positive attitudes toward collaboration  -Supportive institutional policies and procedures  -Designing promotion and tenure and other organizational processes to support interdisciplinary team science  -Planned workshops for coordinating different disciplinary and other knowledge commitments  -Clarify language differences across disciplines/backgrounds  -Collaborate with team members with different working styles  -Background of participants  -Developing organizational structure  -Discovery and verification  - Organizational structures [30, 31, 34, 36, 38, 39, 41, 43-46, 48, 50, 53, 54, 61, 63, 64]  **Sub-theme2:** Team Science skills training  - Team science skills training  - Using mentors (mentoring) for team science  -Training for facilitating team effectiveness/organization  -Building interpersonal competencies among faculty and trainees  - Training for a transdisciplinary research  - Training/ nurturing  -Training interdisciplinary scientists  - Team training model  - Targeting interdisciplinary training  -Identifying mentors to focus on team integration issues  - Training meetings to build readiness  -Team science training  -A partnership-building skill set  -Workshop  - Mentoring team members  -Team learning (training)  - enhancing outcomes by integration of teaching/training, research/discovery, and extension/engagement [31, 35, 36, 41, 44, 47, 56, 62]  **Sub-theme3:** Evaluation  -Formal evaluation processes  -Assessing team performance  - Establish an accountability strategy for evaluation  - Having a plan to assess whether the research team is meeting its goals regarding interdisciplinarity  -Evaluating team members and other project members  -Providing feedback to team members  -Recognition of team member achievements. [32, 44, 48, 60, 63, 64] |
| **Themes 3: Technology**  **Sub-theme1:** Complexity of techniques  - Complexity of techniques  [37]  **Sub-theme2:** Privacy issues  -Data sharing issues  -Regulatory/privacy issues  [37] | **Themes 3: Technology**  **Sub-theme1:** Virtual readiness  [53]  **Sub-theme2:** Data management  [64] |
