# Supplementary figures and images for: Barriers and facilitators of conducting research with team science approach: a systematic review
Source: BMC Med Educ. 2023 Sep 5;23:638. doi: 10.1186/s12909-023-04619-0 (PMC10478305; doi:10.1186/s12909-023-04619-0)

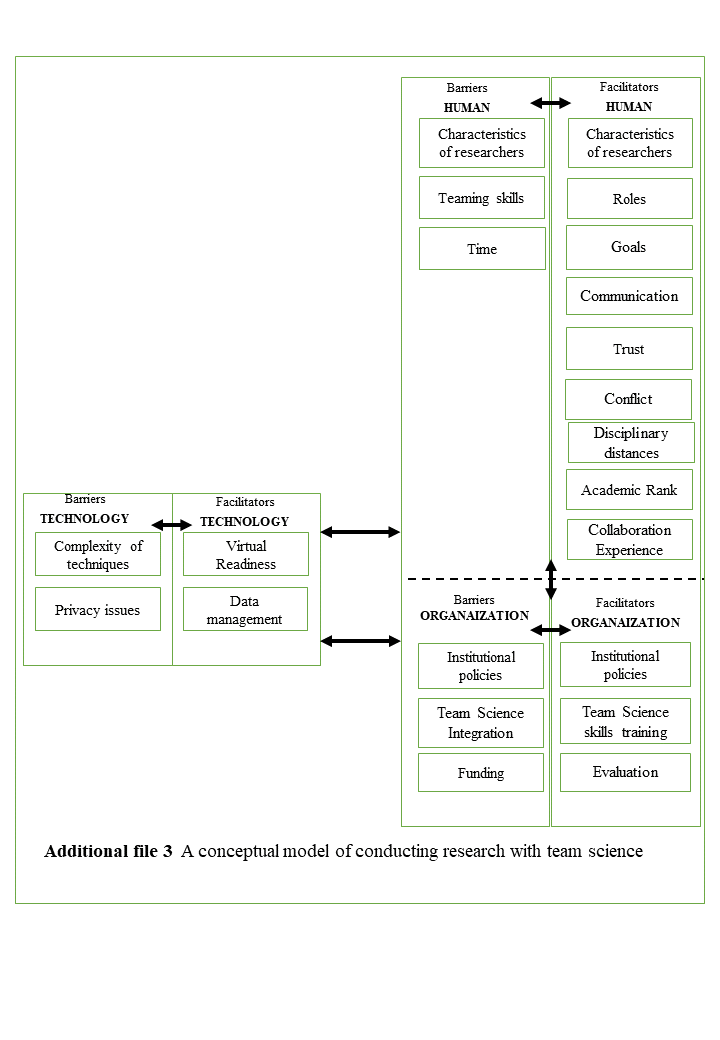

Supplement: Supplementary file 2 — Supplementary Material 2 [file 12909_2023_4619_MOESM2_ESM.png]
